# Utilization of a 3D-Printed Mandibular Jaw for Ridge Reconstruction in Periodontics: A Case Report

**DOI:** 10.7759/cureus.61092

**Published:** 2024-05-26

**Authors:** Badr Othman, Mada K Al-Arfaj

**Affiliations:** 1 Periodontology Department, Faculty of Dentistry, King Abdulaziz University, Jeddah, SAU; 2 Periodontology Department, Prince Mohammed Bin Abdulaziz Hospital, Medina, SAU

**Keywords:** dental implant surgery, ‎3d printing, cone-beam computed tomography (cbct), ridge augmentation, gbr (guided bone regeneration)

## Abstract

Three-dimensional (3D) printing is an emerging manufacturing technology in dentistry with a range of applications. Digital dentistry presented in cone beam CT scan radiographs is a revolution that improved surgical outcomes by optimizing accurate diagnosis and analysis of the surgical sites before surgery. A periodontist can modify the treatment plan, surgical techniques, and incision design based on bone defects seen on cone beam CT scans. Block grafting has been a technique of choice when wound stability is required for guided bone regeneration. There was no significant difference between the different surgical procedures for reconstruction and choice should be given to the simpler and less invasive procedure. A xenograft or allograft block can work as an alternative to the autogenous bone block to reduce the surgery time and patient morbidity. Preparation and shaping of block graft during surgery time to match the defect shape can prolong the operative time, reduce the treatment success, and increase postoperative complications. In this case report, a sterilized 3D-printed mandibular jaw was utilized to visualize the defect size and shape. A bovine xenograft block was then prepared, shaped, and adapted on the 3D-printed jaw 30 minutes before the surgery. The block graft was then transferred and well-fitted on the surgical defect. Handling experience was greater and surgery time and postoperative pain were reduced*.*

## Introduction

The ways of living and working nowadays have been modified by modern technologies [1]. It significantly improved and enhanced the treatment planning and communication process as well as the treatment outcomes with undeniable advantages [2]. Three-dimensional (3D) printing is an emerging manufacturing technology in dentistry with ranged applications in prosthodontics, orthodontics, implants, and periodontics [3]. It allows the dentist to custom design and print implant surgical guides and provisional and orthodontic appliances [4]. Digital dentistry presented in cone beam CT (CBCT) scan radiographs is a revolution that improved surgical outcomes by optimization of accurate diagnosis and analysis of the surgical sites prior to surgery. A periodontist can modify the treatment plan, surgical techniques, and incision design based on bone defects seen on CBCT [5]. Block grafting has been a technique of choice when wound stability is required for guided bone regeneration. There was no significant difference between different surgical procedures for reconstruction and choice should be given to the simpler and less invasive procedure [6]. Utilizing 3D technologies results in numerous advantages. A xenograft or allograft block can work as an alternative to the autogenous bone block to reduce the surgery time and patient morbidity. Preparation and shaping of block graft during surgery time to match the defect shape can prolong the operative time, reduce the treatment success, and increase postoperative complications. Utilization of a 3D-printed jaw produced from autoclavable material allows preoperative manual milling and adaptation of an allogeneic corticocancellous iliac block graft for horizontal ridge augmentation. It improved visualization of the ridge defect, and a significant reduction of intra-operative time when compared to the conventional technique, where most of the time during surgery is spent shaping the bone block and adapting the graft to the recipient site [7]. In this case report, a sterilized 3D-printed mandibular jaw was utilized to visualize the defect size and shape. A bovine xenograft block with a size of 1 × 1 × 2 cm (height × width × depth) from Bio-Oss Geistlich (Wolhusen, Switzerland) was prepared, shaped, and adapted on the 3D-printed jaw 30 minutes prior to the surgery and then stored in a sterile environment. The block graft was then transferred, well-fitted, and adapted to the exact size and shape of the surgical defect. Tissue handling experience was greater and surgery time and postoperative pain were reduced.

## Case presentation

A 30-year-old, medically fit, female patient presented to the clinic with a chief complaint to replace her missing tooth in the lower left side that had been extracted a long time ago. Upon clinical and radiographical examination, there was a missing lower left 1st molar (tooth #36) with vertical and horizontal ridge deficiency. The case was treatment planned for guided bone regeneration utilizing a block graft, followed by a dental implant four to five months later and to be restored as a single implant crown #36.

Pre-surgical planning

An alginate impression was taken and poured into a study model. A diagnostic wax-up was made to fill tooth #36 and then duplicated into a study model. A scanning appliance was fabricated from a thermoplastic stent on top of that study model, and then filled with radiopaque material at the tooth #36 site.

**Figure 1:**
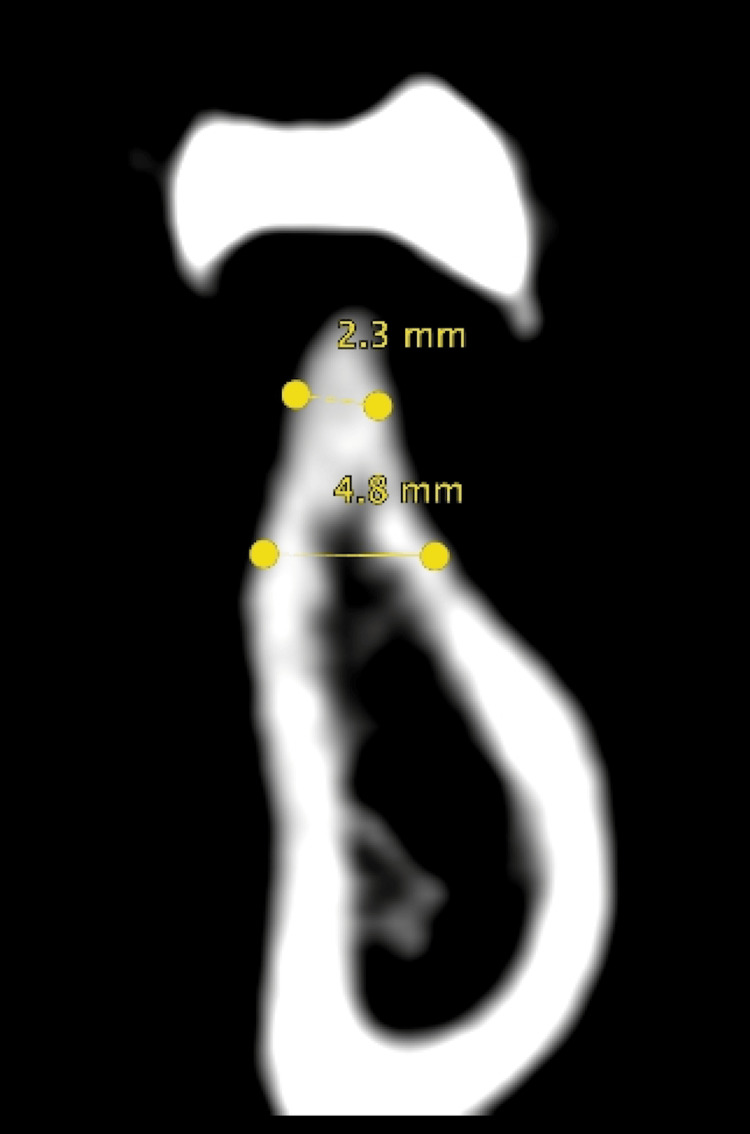
Cone beam CT analysis.

A CBCT scan was taken while the patient was wearing the scanning appliance. CBCT analysis showed 2.30 mm horizontal bone at the crest, 4.77 mm at the middle, and 12.73 mm vertical bone from the crest to the inferior alveolar nerve (IAN) canal (Figure 1).

**Figure 2:**
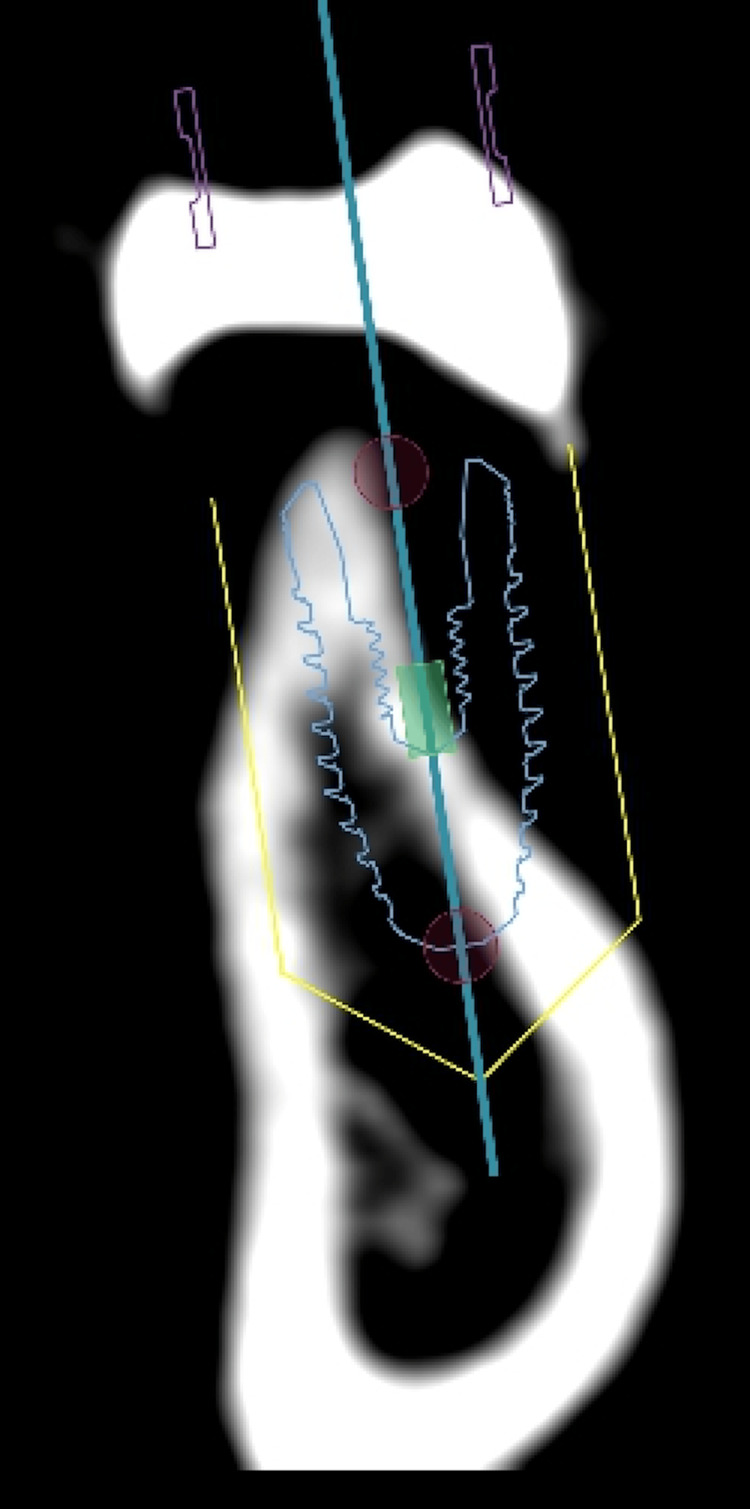
Planning to place the dental implant.

After consultation with the prosthodontics department, the case was treatment planned for guided bone regeneration utilizing a xenograft block graft, followed by a dental implant four to six months later and to be restored as a single implant crown #36 (Figure 2).

**Figure 3:**
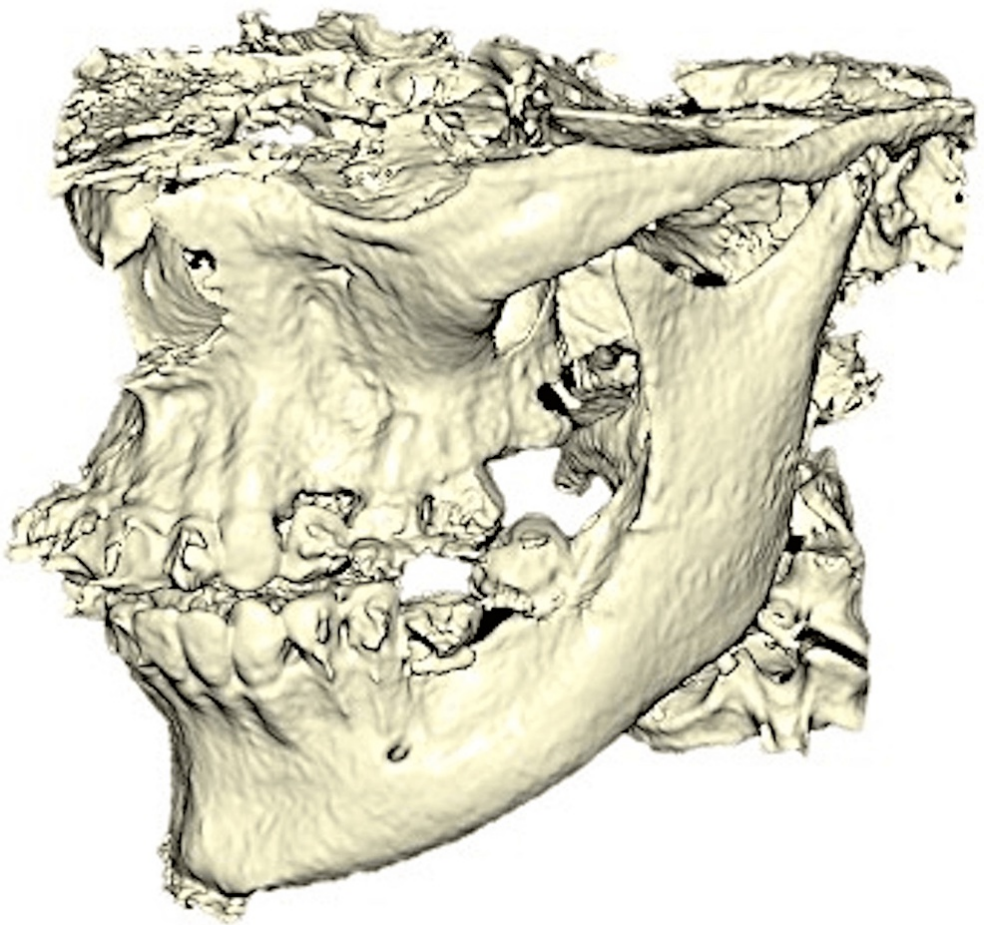
Mandibular jaw (cone beam CT) image was converted into an STL file.

CBCT mandibular jaw image was converted into an STL file via Blue Sky Plan v4 (Blue Sky Bio, Libertyville, IL) software (Figure 3).

**Figure 4:**
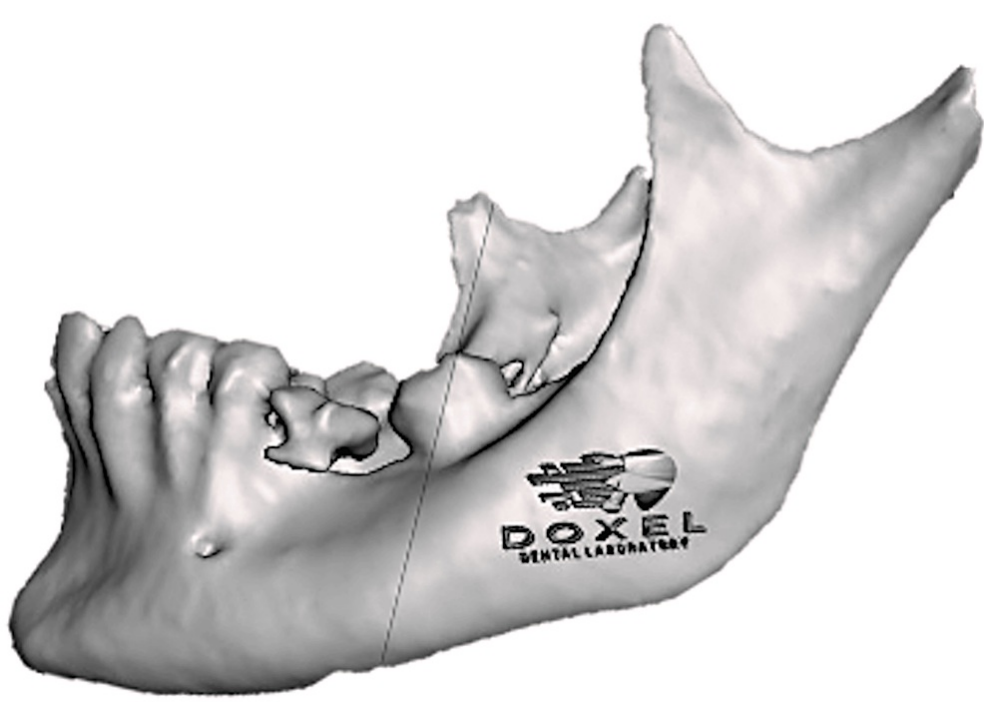
Refined mandibular jaw in the STL format.

The STL file was imported into Meshmixer (Autodesk, San Francisco, CA) to separate the mandible from the maxilla and refine it (Figure 4).

**Figure 5:**
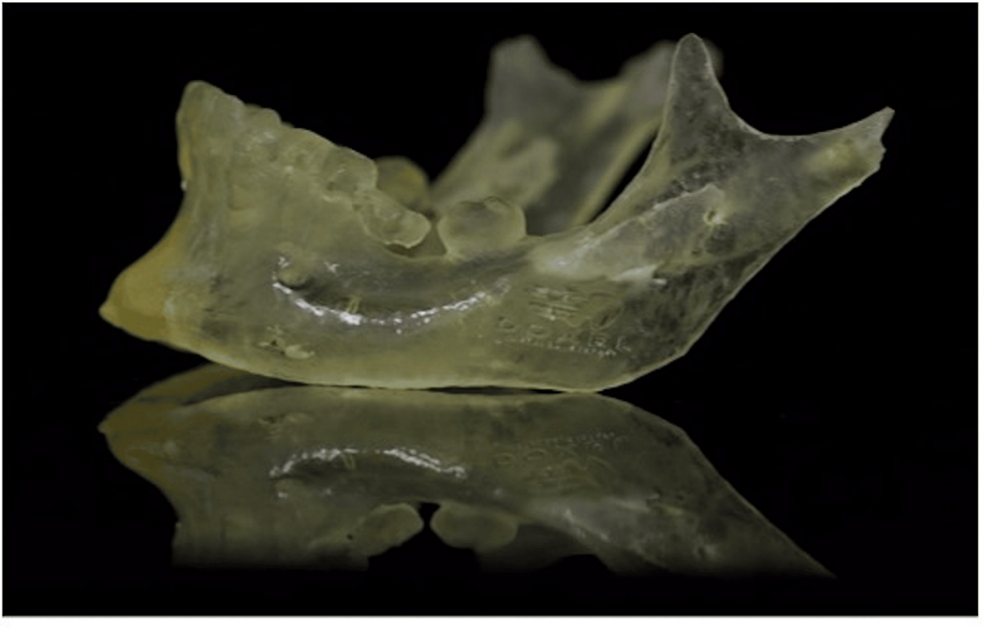
A 3D-printed mandibular jaw.

A 3D-printed mandibular jaw was constructed in an autoclavable nylon polyamide thermoplastic material (Figure 5).

**Figure 6:**
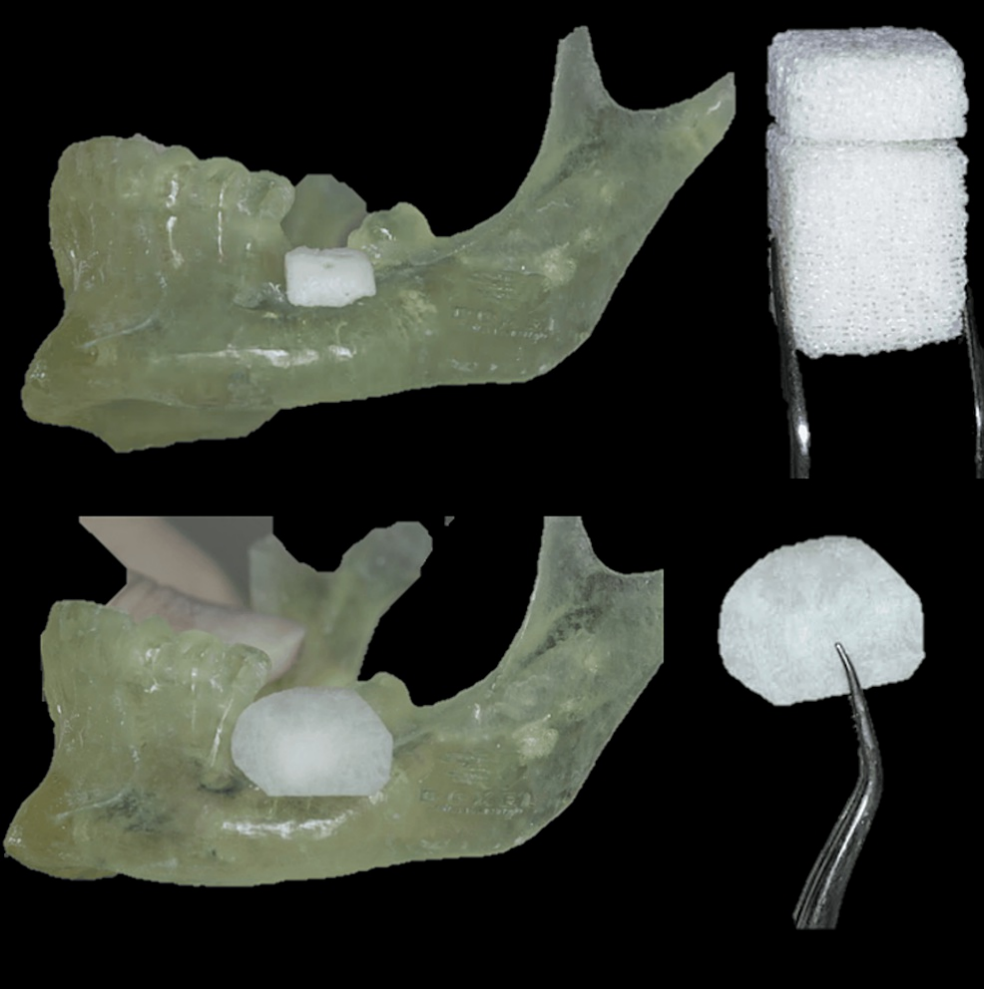
Pre-surgical preparation of the bone graft and membrane.

The jaw was sterilized. A bovine xenograft block with a size of 1 × 1 × 2 cm (width × depth × height) from Bio-Oss Geistlich was hydrated in normal saline and then bone block and membrane were prepared, shaped, and adapted on the printed jaw to fit the defect. A fixation screw hole was prepared (Figure 6).

Surgical procedure

**Figure 7:**
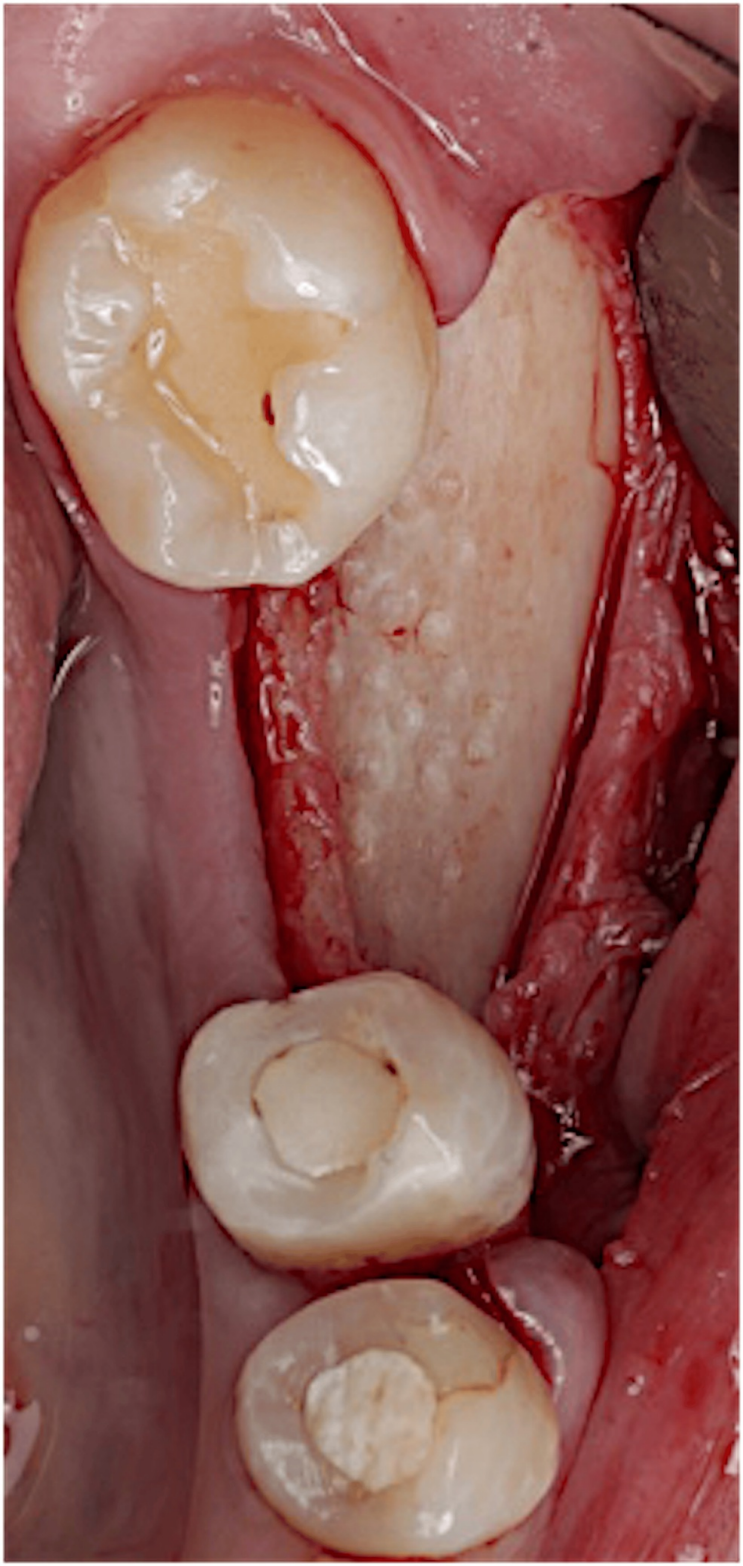
Flap reflection.

The patient presented to the clinic after the bone block and membrane had been prepared and ready to be used. Anesthesia was given to the patient (buccal and lingual infiltrations using lidocaine hydrochloride 2% and adrenaline 1:80,000, two carpules). Midcrestal incision at the area of tooth #36, with sulcular incisions around #34 and #35 and vertical releasing incisions mesial to #37 were made. A full-thickness mucoperiosteal flap was reflected exposing the buccal bone, and a periosteal-releasing incision was done to relieve and prepare the flap for coronal advancement and proper closure (Figure 7).

**Figure 8:**
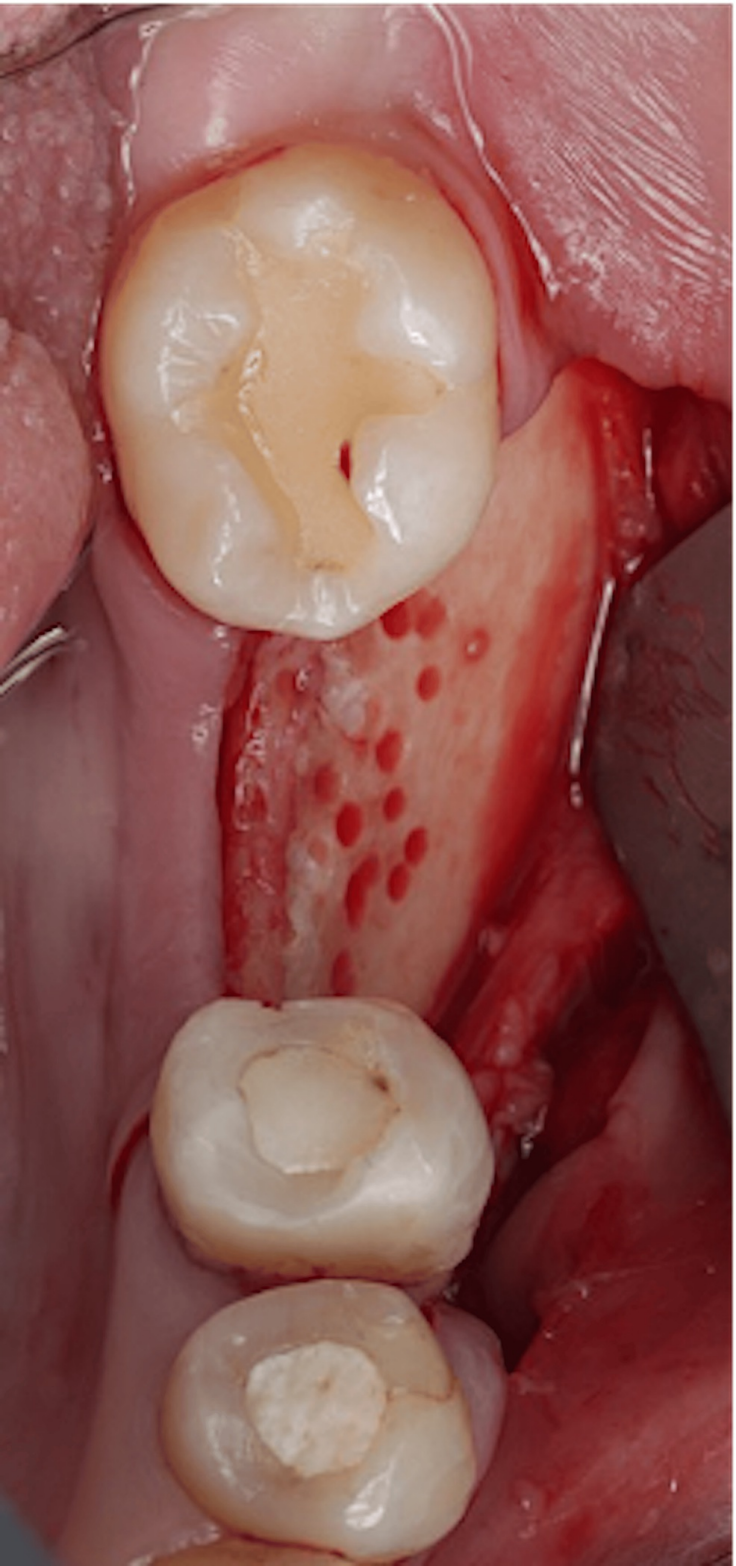
Decortication.

Decortications were created at the recipient site using a small round diamond bur (Figure 8).

**Figure 9:**
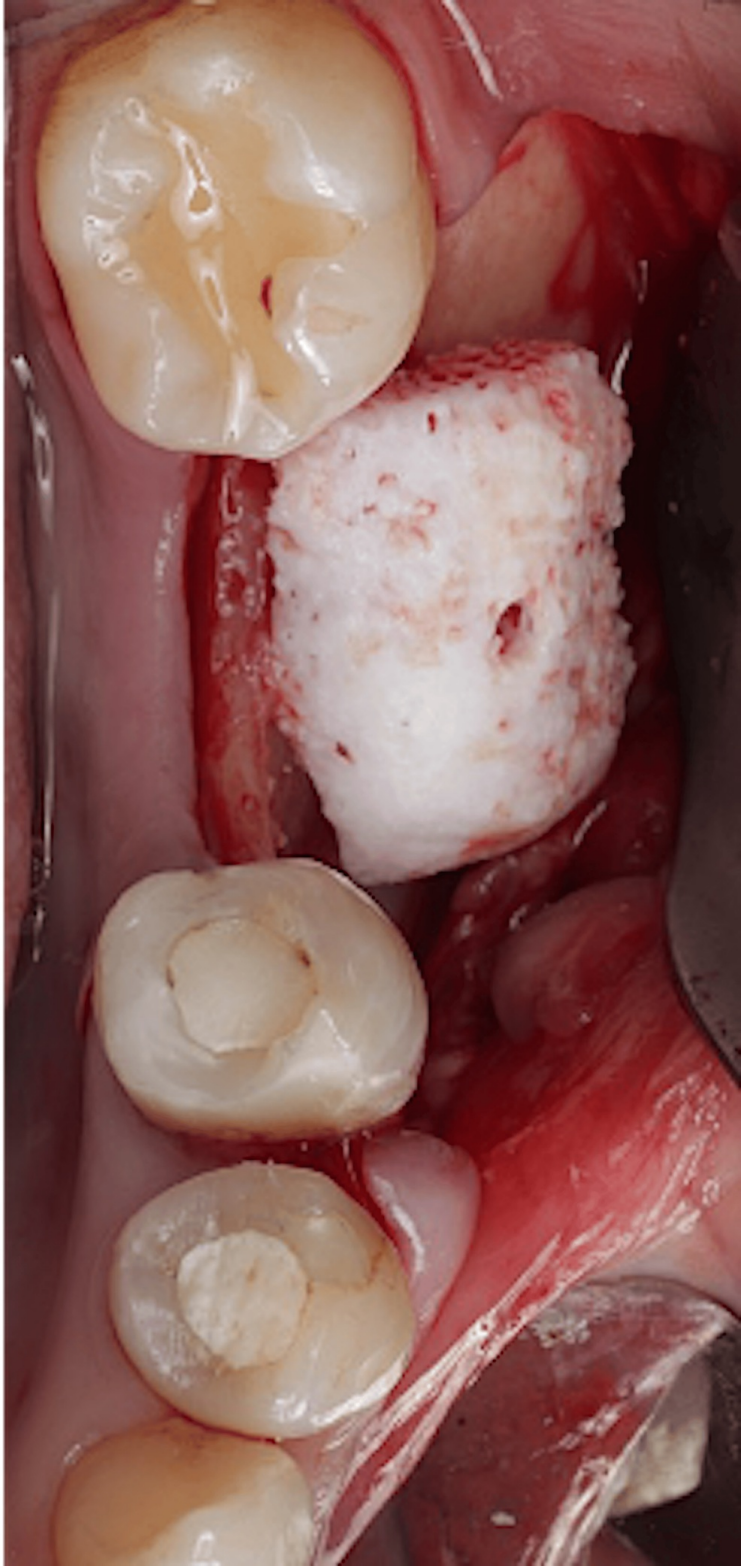
Block adaptation.

The bone block was transferred intraorally, and it was checked for adaptation and fitting, which was precise and remarkable. A fixation screw hole was prepared at the recipient buccal site and the block was stabilized with one fixation screw (size 1.4 x 10 mm) (BioHorizons fixation screw kit, Birmingham, AL) (Figure 9).

**Figure 10:**
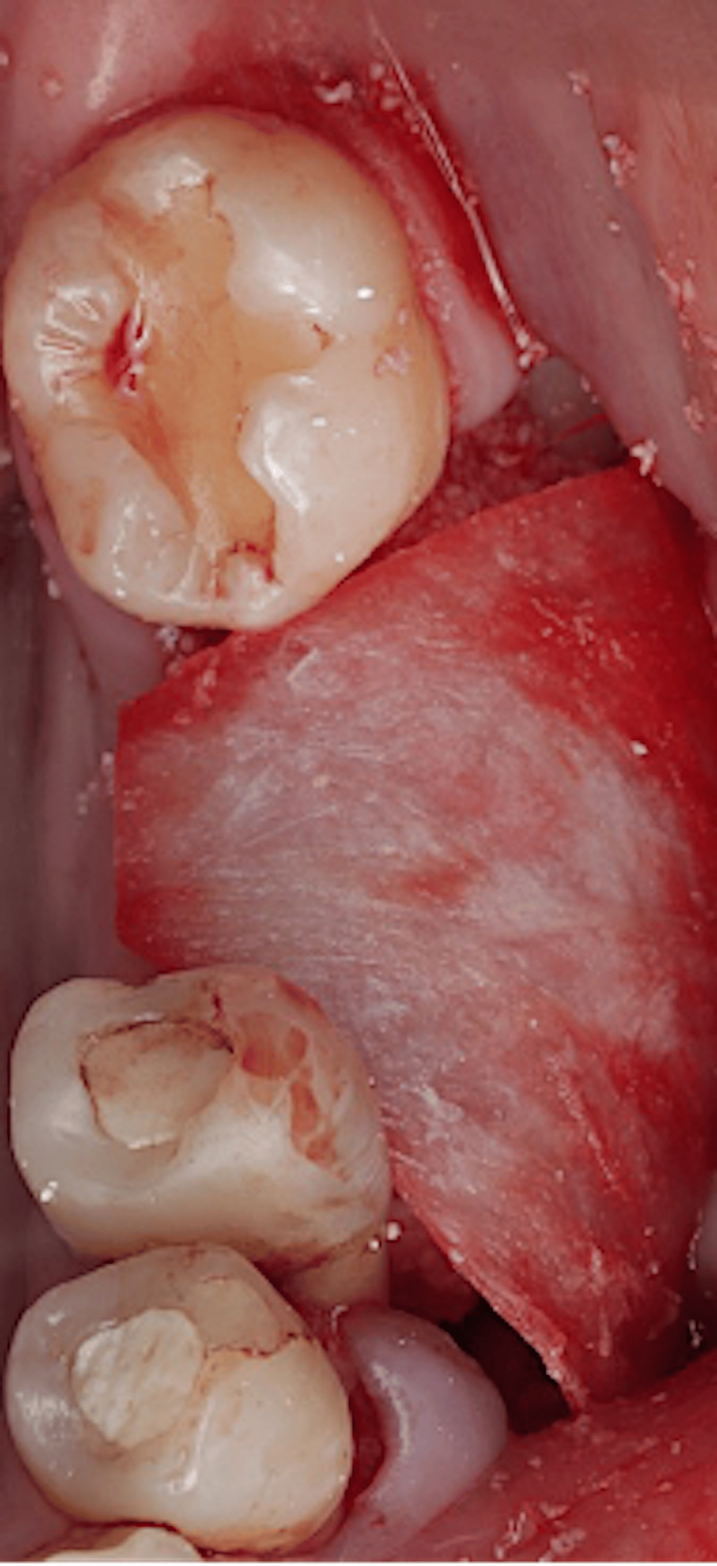
Membrane adaptation.

Particulate xenograft material was placed over the block and defect. A collagen membrane (Citagenix, Laval, Canada) of 30 x 40 mm was placed over the grafted site (Figure 10).

**Figure 11:**
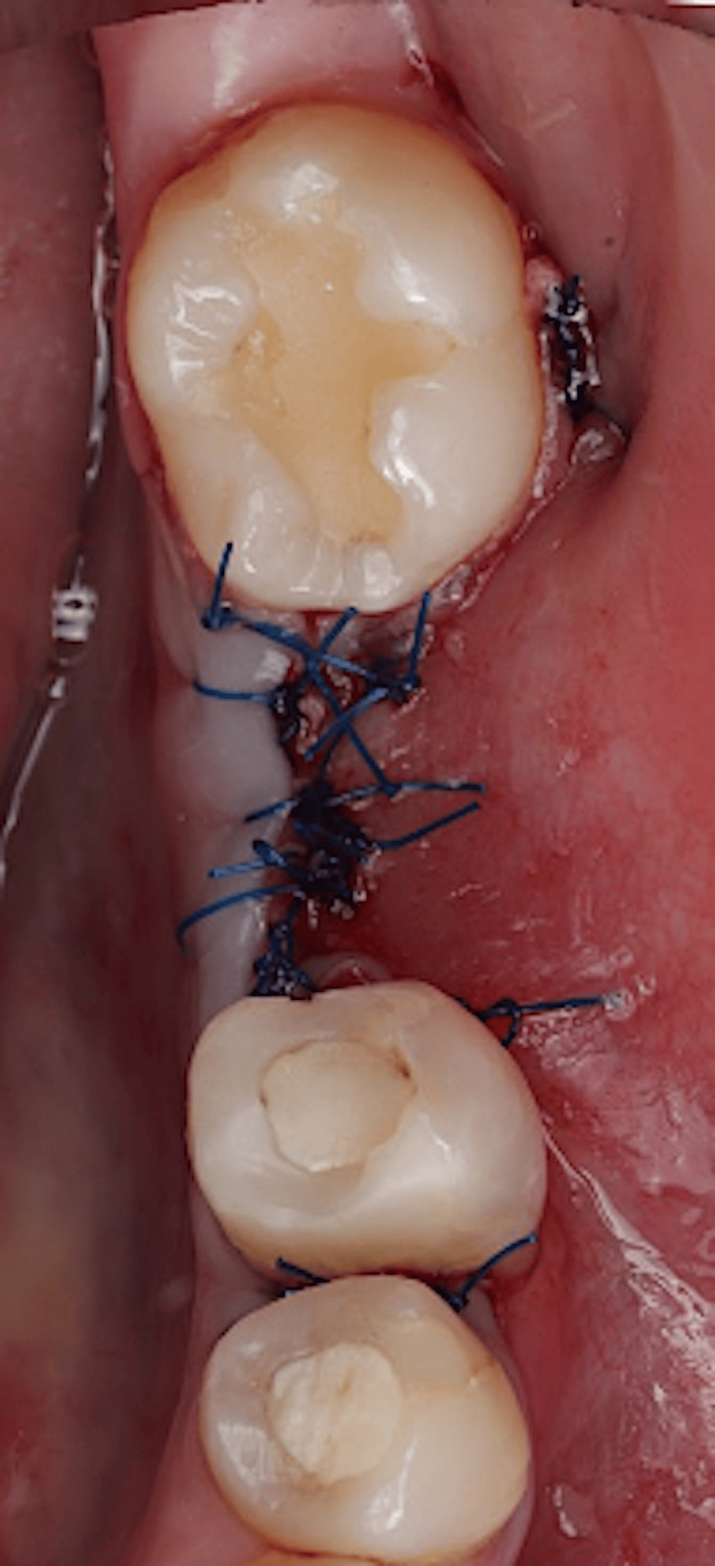
Flap closure.

The flap was re-approximated and sutured with passive primary closure (Figure 11).

**Figure 12:**
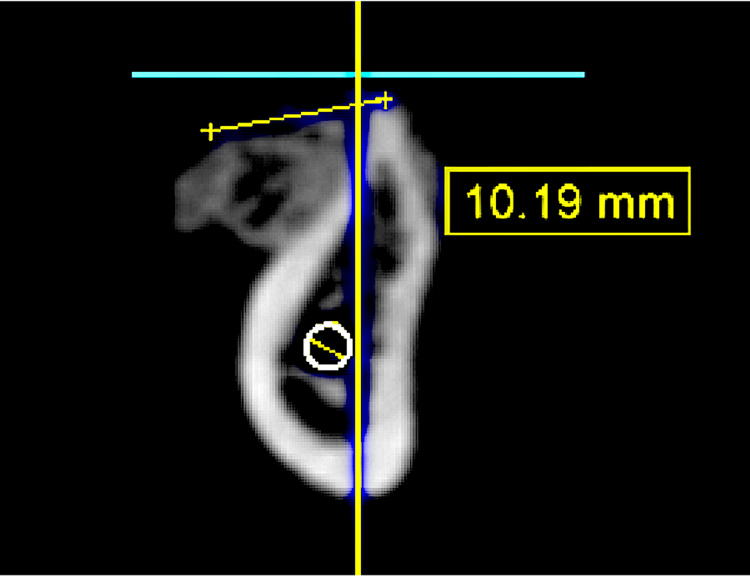
Cone beam CT cross-section to show the grafted site after six months of the surgery.

CBCT was taken after six months of healing to evaluate the amount of bone gain at the cross-section of the site (Figure 12).

**Figure 13:**
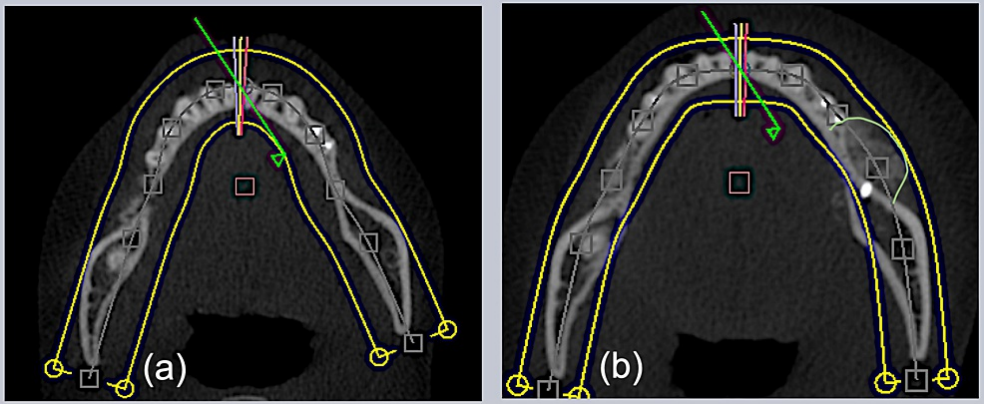
Cone beam CT occlusal view comparison. (a) Before; (B) after.

CBCT was taken after six months of healing to evaluate the amount of bone gain at the occlusal view of the site (Figure 13).

**Figure 14:**
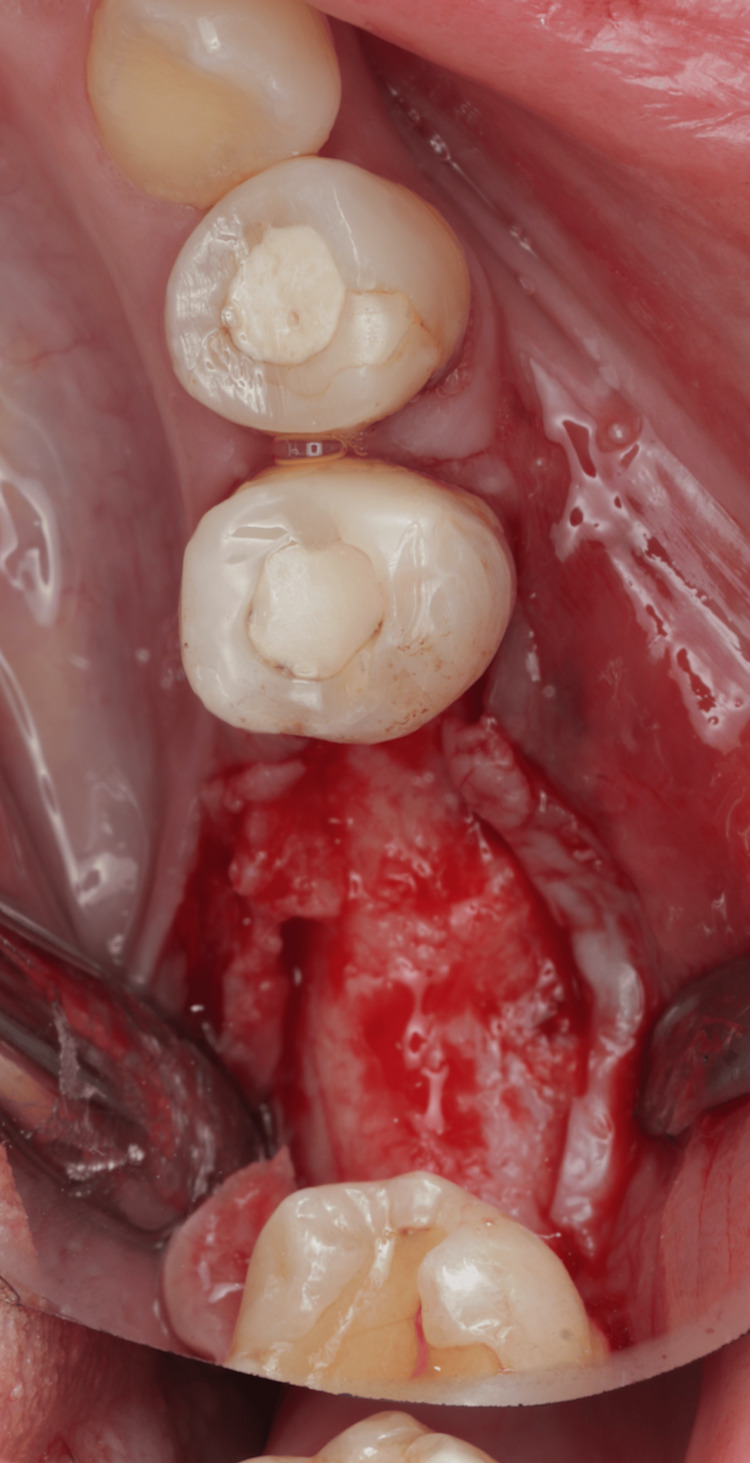
Surgical site at six months re-entry.

A full mucoperiosteal flap was reflected on the surgical site to re-enter and place the dental implant on the grafted site. The bone width was significantly gained (Figure 14).

**Figure 15:**
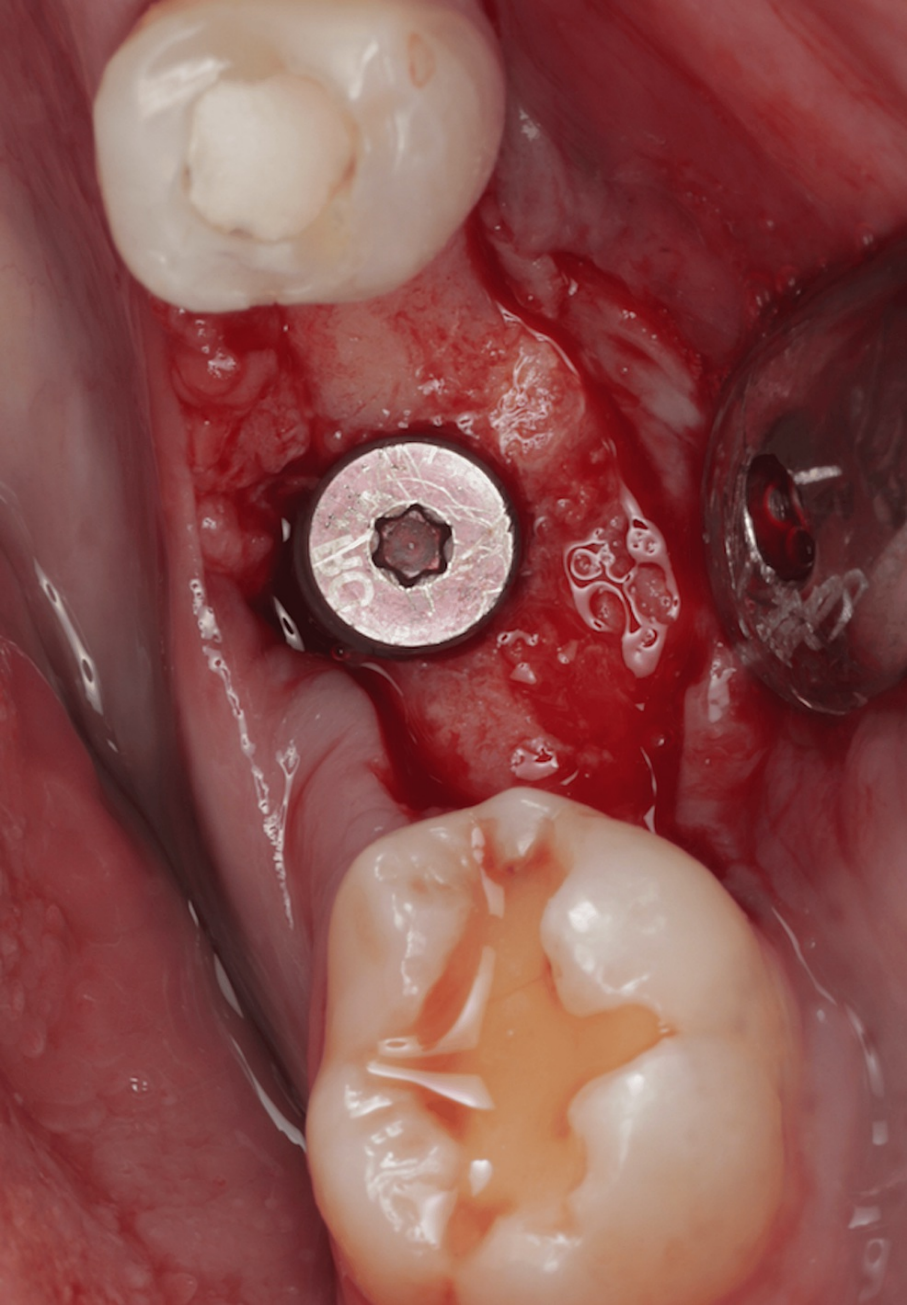
Dental implant was successfully placed with high stability.

The osteotomy was created and the dental implant (Bone Level Tapered Implant (BLT) 4.1 x 10 mm, Straumann, Basel, Switzerland) was successfully placed with high stability (Figure 15).

## Discussion

Technological development has considerably changed the paradigm of implantology, providing dentists with multiple new opportunities and tools for replacing teeth and improving other dental surgery outcomes. Digital data acquisition for planning procedures, advanced surgical techniques based on optical coherence and CBCT as well as artificial intelligence, and many other tools have appeared. Moreover, entirely new systems have been developed, including those for the simultaneous interaction of digital planning with surgical and prosthetic procedures, and suitable dental materials, resulting in essentially fast, reliable rehabilitation, thanks to implants, and a new personalized dental medicine era. Despite the above-mentioned progress, there is significant value in the implementation of new technologies while focusing on the need to ensure the best possible patient outcomes [8]. Clinicians can monitor the 3D anatomy and quality of bone due to the recent developments in computer hardware and software, which aids in the diagnosis of reparative or augmentative grafting to develop favorable osseous conditions for implant placement. Advanced synthetic bioactive resorbable bone biomaterials such as the cluster-like, hydrophilic, particulate graft have also allowed the modification of the existing bone topography to maintain cofferdam levels, facilitate ridge preservation, augmentation, or enhancement to achieve successful prosthetic rehabilitation by bridging lost bone anatomy and aiding in implant placement using its specific clinical handling features and case applications [9]. The high variability of radiation doses and diagnostic image quality of dental CBCT machines requires prior optimization and justification before clinical use. Since the equivalent doses and image quality range extensively between machines and exposure protocols, it is vital to undertake such procedures. Nevertheless, some challenges in the dental CBCT method include segmentation accuracy and patient-related factors, such as motion or metal artifacts. Nonetheless, this modality has a high potential for usage in the presurgical diagnosis, planning, and transfer for oral implant rehabilitation, and requires optimization based on machine-dependent, patient-specific, and indication-oriented variables [5]. 3D planning allows the construction of precisely 3D surface models, dynamic cephalometry, bone cutting, and the repositioning thereof. The system is used to provide different stages of intraoperative guidance, via the positioning splints and the real-time positioning regarding the jaw and 3D in due portions. The same has a positive impact on the patients and the practice of the surgical field, and it is set to provide more detailed operative plans, with precision and better case documentation [10]. 3D radiological displays, such as CBCT, are commonly used for precise diagnosis of bone pathology and complicated implant-supported prosthetic restorations. The safety and simplicity of use in dental practices frequently allow for clinical indications to be covered. CBCT utilization in the Planmeca Promax 3D trial was most prevalent in oral surgery and implantology referral, allowing for various questions to be addressed. They can range from wisdom tooth anatomy to impacted canines, cystic lesions, and, in some situations, maxillary sinus issues, which help to plan for surgery and digital therapy preparation to be especially well-engineered [11]. The CBCT in periodontology was also a topic of a systematic literature review. It showed good CBCT accuracy for the visualization of the periodontal structures and positive impact during the regenerative surgery for periodontal defects [12]. After comparison of CBCT to conventional methods for furcation assessment in the first maxillary molar, it can be concluded that there were statistically significant differences in the diagnosis of furcation using CBCT, which generates more information and is, therefore, a more reliable base than conventional methods [13]. Due to the growing capabilities of imaging and modeling technologies, there is an increase in the relevance of 3D printing for dentistry and maxillofacial surgery. Moreover, the following processes allow the creation of drill guides to make the process of implantation more precise, and create physical models for prosthodontics and surgery, and dental and orthopedic implants [14]. The application of 3D printing technologies in dentistry, namely, prosthodontics, oral surgery, and implantology, is increasing and giving insight into the probable ways of the implementation of new materials and technologies, indicating the high potential possibility of 3D printing in dentistry [3]. In another review, the history and usage of 3D printing technologies in dentistry, which regularly extends prosthodontics, oral surgery, and implantology was analyzed. Most prevalent printing methods, essential factors that influence their metric, utilities such as high material use, and limitations such as high expenses and post-processing duration are well presented. The trends foresee the significant role played by new materials and technologies, which demonstrates a bright future for 3D printing in dentistry [15]. 3D visualization is likely to facilitate the surgical procedure and might potentially reduce the incidence and/or severity of most adverse events [16]. In another review, the work performed on oral and maxillofacial surgery and the musculoskeletal system was analyzed. Despite the reduction in operating time and increased accuracy of 3D-printed devices, their effectiveness in different medical fields has not been established. Consequently, their manufacture remains a candidate for rigorous verification before finally establishing in clinical practice [17]. Reviewing human clinical trials comparing flapless and conventional flap techniques for implant placement, there was no significant difference in survival rates, marginal bone loss, or soft-tissue parameters when proper protocols were followed, and performed by an experienced surgeon. A decrease in surgical time and increased patient comfort and acceptance were achieved with flapless procedures, as opposed to more prolonged flap surgeries [18]. A mechanical device and scanning template were utilized in this image-guided implant placement system study to assess the system’s reliability. The preoperative planning coincided with the intraoperative results highly. Thus, the image-guided implant placement system has the potential as a reliable device to evaluate implant size and anatomic complications. It will be beneficial with flapless surgery [19]. A 37-year-old female patient had a dental implant placement procedure with an allograft due to severe ridge resorption. In this case, the use of the allograft expanded the ridge, and osseointegration of the implant was successful. Since the allograft was successful, it may be utilized as an alternative to autogenous bone grafts, necessitating fewer surgical sites [20]. To conclude, the present case report and subsequent discussion shed light on the transformative effect brought by recent technological advances in the field of implantology. The use of innovative methods for planning and conducting dental operations is affected by several unprecedented innovations such as digital data collectors, cutting-edge surgery techniques, and 3D printing. Nonetheless, this should not prevent the use of the possible level of technologies on behalf of patients and their well-being, distracting the challenges like an unequal amount of radiation dosage and the quality of diagnostic images.

## Conclusions

The use of 3D printing technology in guided bone regeneration has revolutionized the pre-surgical planning and designing of block grafts. The integration makes the processes easier, faster, and more accurate than ever. Since the process is designed to enable meticulous planning and customization of grafts, 3D printing makes the process take less time, thereby reducing the risk of post-surgical complications.
